# Weed presence altered biotic stress and light signaling in maize even when weeds were removed early in the critical weed‐free period

**DOI:** 10.1002/pld3.57

**Published:** 2018-04-23

**Authors:** David P. Horvath, Stephanie Bruggeman, Janet Moriles‐Miller, James V. Anderson, Munevver Dogramaci, Brian E. Scheffler, Alvaro G. Hernandez, Michael E. Foley, Sharon Clay

**Affiliations:** ^1^ Sunflower and Plant Biology Research Unit USDA/ARS/RRVARC Fargo North Dakota; ^2^ Plant Science Department South Dakota State University Brookings South Dakota; ^3^ University of South Dakota Sanford School of Medicine Internal Medicine Department Sioux Falls South Dakota; ^4^ Genomics and Bioinformatics Research Unit USDA/ARS Stoneville Mississippi; ^5^ Roy J. Carver Biotechnology Center Urbana Illinois

**Keywords:** maize, plant–plant interaction, transcriptome, weeds

## Abstract

Weed presence early in the life cycle of maize (typically, from emergence through the 8 to 12 leaf growth stage) can reduce crop growth and yield and is known as the critical weed‐free period (CWFP). Even if weeds are removed during or just after the CWFP, crop growth and yield often are not recoverable. We compared transcriptome responses of field‐grown hybrid maize at V8 in two consecutive years among plants grown under weed‐free and two weed‐stressed conditions (weeds removed at V4 or present through V8) using RNAseq analysis techniques. Compared with weed‐free plant responses, physiological differences at V8 were identified in all weed‐stressed plants and were most often associated with altered photosynthetic processes, hormone signaling, nitrogen use and transport, and biotic stress responses. Even when weeds were removed at V4 and tissues sampled at V8, carbon: nitrogen supply imbalance, salicylic acid signals, and growth responses differed between the weed‐stressed and weed‐free plants. These underlying processes and a small number of developmentally important genes are potential targets for decreasing the maize response to weed pressure. Expression differences of several novel, long noncoding RNAs resulting from exposure of maize to weeds during the CWFP were also observed and could open new avenues for investigation into the function of these transcription units.

## 1 INTRODUCTION

Weeds are known to reduce crop biomass and grain yield. The most discussed mechanism underlying these phenomena is resource competition for light, nutrients, and/or water. Validation of the competition hypothesis in field studies has proven elusive (Carlson & Hill, 1985; DiTomaso, 1995; Young, Wyse, & Jones, 1984). The time when crops are most sensitive to weed presence is during early to mid‐establishment (the critical weed‐free period [CWFP]) (Zimdahl, 2007), and weeds can impact season‐long yield even if removed after the CWFP (Knezevic, Evans, Blankenship, Van Acker, & Lindquist, 2002; Page et al., 2012). Additionally, in most modern agricultural systems, resources such as water, nutrients, and light are rarely limiting during the CWFP; a result of fertilizer being applied, spring rains providing an abundance of water for seedling crops, and abundance of light for small plants is greater than needed. After weed removal, crop plants never fully recover, even if supplied with greater abundance of the previously mentioned resources. This irreversible response would not be expected if weeds primarily reduced yield by reducing resource availability. Rather, this suggests that weed presence alters crop physiology or development (Zhu, Vos, Van der Werf, Van der Putten, & Evers, 2014) such that the negative growth response persists even if weeds are removed during or shortly after the CWFP.

Further evidence that resource competition is not the sole mechanism of weed–crop interactions come from studies demonstrating that weeds alter maize (*Zea mays* L.) development even when physically separated at the soil level, and no light competition is possible (Liu, Mahoney, Sikkema, & Swanton, 2009). In the Liu et al. (2009) study, maize and weeds were grown in separate, but adjacent pots, or maize was grown alone. Plants grown adjacent to weeds had reduced leaf area, biomass, and yield and displayed the same characteristic responses as maize grown under weed‐stressed field conditions (Liu et al., 2009). Follow‐on studies implicated a possible role for light quality—at least in responses of maize seedlings (Afifi & Swanton, 2011; Page, Tollenaar, Lee, Lukens, & Swanton, 2009). Maize seedlings given lower ratios of red (R) to far‐red (FR) light (R:FR) presented many of the characteristics associated with weed presence. Based on results from these studies, it has been hypothesized that maize detects weeds (or other nearby plants) because light reflected from plants is higher in FR and lower in R light compared with “normal” light. The change in the R:FR ratio is thought to be perceived by maize plants in a manner similar to the shade avoidance signaling process that has been well characterized in the model plant arabidopsis (*Arabidopsis thaliana* Heyn). However, not all responses generated by weed presence were manifested in response to lower R:FR ratios. Interestingly, many of the deleterious responses of maize to weeds could be alleviated by pretreating the seedlings with a fungicide known to impact the oxidative stress response in maize seedlings (Afifi, Lee, Lukens, & Swanton, 2015).

Other studies suggest that the response of maize to weeds may be more complicated than simple induction of the shade avoidance response (Afifi & Swanton, 2012). Microarray studies done to determine the response of maize to weeds, shade, or low nitrogen (Moriles et al., 2012) indicated that some of the responses to these diverse stresses were similar and some were not. For example, the impact of these stresses on expression of genes encoding components of the photosynthetic apparatus indicated there were 338 genes differentially expressed relative to the weed‐free control that were specific to weed stress (Moriles et al., 2012). Also, while transcriptome studies have strongly implicated shade avoidance as a component of soybean response to weeds during the CWFP (Horvath et al., 2015), shade avoidance responses were not as strongly implicated in similar transcriptomic studies of maize (Horvath, Gulden, & Clay, 2006). Likewise, a comparison of the transcriptomic response of maize growing under high planting density compared to maize growing in response to weed pressure, indicated that there were differences in how maize responded to intra‐ and interspecies competition (Clay et al., 2009; Moriles et al., 2012). However, in all of these microarray analyses, there appeared to be a high degree of false positives as indicated by the relatively high number of genes that failed to show consistent gene expression patterns when examined by qRT–PCR (Moriles et al., 2012). In most cases, such differences were attributed to inability to distinguish gene family members or alternate splicing of transcripts, which would be indistinguishable on the cDNA microarrays used for most of these analyses.

Recent developments in next‐generation sequencing offer the possibility for more precise transcript analysis. RNAseq produces sequence reads directly from cDNAs and these sequences can be assembled de novo to provide full‐length and partial transcript sequences, or be matched (mapped) to annotated exons of known or suspected genes in fully sequenced genomes, such as those available for maize (Schnable et al., 2009). Because the number of sequences generated from any given transcript is stoichiometric to the number of cDNAs in the original library, the expression of any given transcript can be determined by simply counting the sequences that exactly match it. Further, various statistical processes have been developed to allow assessment of expression when sequences match two or more transcripts in cases where transcripts from paralogous or alternately spliced genes share high sequence identity (Kim et al., 2013).

**Figure 1:**
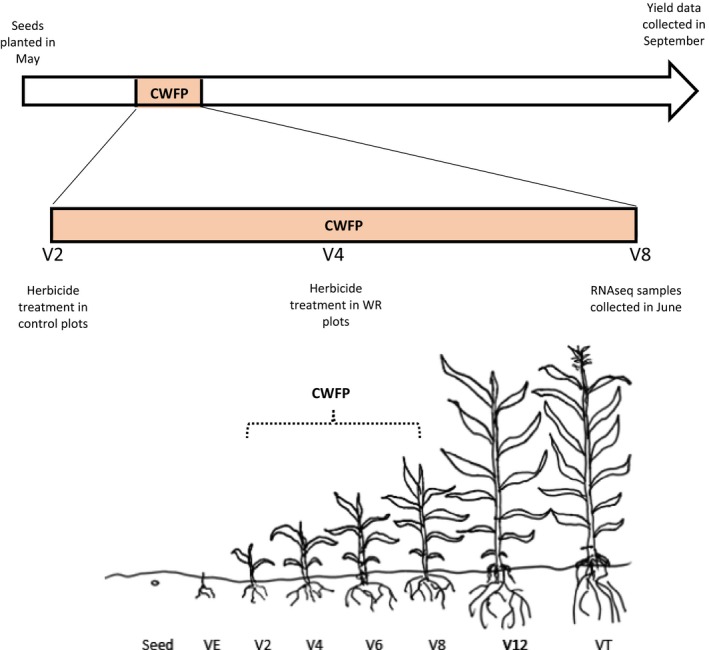
Maize seeds were planted in May of 2007 and 2008. The weed‐free control plots were treated with herbicides for weed control prior to the critical weed‐free period (CWFP) at V2 and again several weeks prior to V8, WR4 plots were treated at the V4 stage for weed removal at that stage; no herbicide was applied to weedy (WR8) treatments until after data and plant tissue collection at V8. All plant material used for construction of RNAseq libraries for weed‐free control, WR4, and WR8 treatments were collected at the V8 stage of maize development (in June for 2007 and July for 2008). Data for yield and biomass production were collected at the end of the growing season (September of 2007 and 2008)

Identification of biochemical pathways and biological/developmental processes that are differentially regulated in response to early‐season weed presence, and that irreversibly impact yield, would be of considerable interest to plant breeders seeking to improve stress tolerance in elite maize hybrids. This understanding may lead to novel weed control mechanisms or, alternatively, manipulation of crop genes to dampen signaling reception of weed presence, thereby reducing negative weed impacts so long as they are removed before any direct competition for resources can occur. Here, we used RNAseq to examine the transcriptome changes at V8 that were manifested in maize when weeds were present through V8, or in recovering plants, when weeds had been removed early in the CWFP at V4 (Figure 1). Gene set and subnetwork enrichment analyses also assisted in understanding relationships among genes and processes affected by weed stress.

## 2 MATERIALS AND METHODS

### 2.1 Plant material for phenological and yield studies

Field experiments were conducted at Aurora, South Dakota (longitude and latitude 96°40′ west and 44°18′ north, respectively). The soil parent materials were loess over glacial outwash, and the soil series was a Brandt silty clay loam (fine‐silty, mixed, superactive, frigid Calcic Hapludolls). The surface horizon contained approximately 110 g sand, 580 g silt, and 310 g clay/kg. Total nitrogen in the 0–15 and 15–60 cm depths were approximately 5.1 and 10.2 mg N/ha, respectively. Total C in the 0–15 and 15–60 cm depths was approximately 44.6 and 78.5 mg/ha, respectively. N‐rate application of 236 kg/ha (based on SDSU soil test recommendations for a yield goal of 13,000 kg/ha) was applied to all plots, and supplemental water was added as needed. Irrigation was applied in four applications of about 2.5 cm each in 2007 and 2008, totaling approximately 10 cm each year. When compared to the 30‐year normal, accumulated growing degree days (GDD, calculated using the 86/50 system) from planting to sampling at V8 were higher in 2007, and similar to the norm in 2008 (Table 1). Precipitation levels in 2007 and 2008 were similar to the 30‐year norm; however, weather immediately prior to sampling at V8 was normal in 2007, but hotter than normal in 2008 (Table 2).

**Table 1:** Accumulated growing degree days (GDD) and precipitation amounts (cm) for each time frame after planting until sampling (Planting to V8), from sampling until harvest (V8 to Harvest) and season long (Total). Data created utilizing https://climate.sdstate.edu degree day tool

Year	Planting to V8		V8 to Harvest		Total	
	GDD	Precip (cm)	GDD	Precip (cm)	GDD	Precip (cm)
2007	574	14	948	16	1,516	30
2008	447	19	843	20	1,290	40
30 year norm	441	15	679	24	1,119	39

**Table 2:** V8 sampling date high and low temperatures, with previous day and week's precipitation and temperature

Year	Temperature (C)				Precip (cm)		
	Sample date high (norm)	Sample date low (norm)	Prior day high (norm)	Prior day low (norm)	Prior 7 day	Season	Prior 7 day
GDD (base 10°C)							
2007	30	15	27	19	0	14	71
2008	29	17	30	18	0	19	75
30 year norm	28	15	27	15			

For 2007 and 2008 experiments, a commercially available 97‐day maize hybrid that had glyphosate resistance and maize rootworm (*Dibrotica virgifera virgifera*)/maize borer (*Ostrinia nubilalis*)‐resistant stacked traits was planted on May 1, 2007, or May 7, 2008, at a seeding rate of approximately 79,000 seeds/ha, with row spacing of 76‐cm. Plants were grown under weed‐free conditions, grown with weeds until V4 (4‐leaf vegetative growth stage) (Nleya, Chungu, & Kleinjan, 2016) when weeds were removed (recovering, or WR4), or grown with weeds through V8 (weed‐stressed, or WR8). Following collection of tissue and phenological measurements at V8, weeds were removed and all plots were maintained weed free until the end of season at which time yield measures were taken. For 2007, the predominant weed was velvetleaf (*Abutilion theophrasti*) with a population between 34–60 weeds/m row. In 2008, another broadleaf, canola (*Brassica napus*), was drilled 10 cm from the maize row at 175 seeds/m^2^ at maize planting to provide a more uniform “weed” density (anticipating a 55% emergence rate, or 96/m^2^ average weed density). Weed‐free control plots were maintained using applications of Dual II Magnum (S‐metolachlor; Syngenta, Greensboro, NC) at 1.6 kg/ha and 1.5 kg/ha prior to the V2 stage of the maize on May 3, 2007, and May 9, 2008, respectively, and Roundup WeatherMax (glyphosate; Monsanto, St. Louis, MO) at 2.24 kg/ha on June 8, 2007, and June 5, 2008. For weed removal at V4, WR4 plots were treated with Roundup Weathermax at 1.26 kg/ha on June 18, 2007, and June 17, 2008, respectively. Weed‐stressed plots were not treated with herbicides. Weeds appearing after herbicide applications were mechanically controlled in all plots using a hoe and hand pulling after data and sample collection at V8 until harvest. A randomized complete block design was used with four replications. Year and block were random effects, whereas treatment (Control, WR4, WR8) was the fixed effect. Environmental differences, herbicide choices, and weed species were varied between years and only differences that were consistent in both years were considered so identification of transcriptome differences resulting from nontarget treatment effects would be limited. Plots within each block were 8 rows wide and 5 m long. Plots were the experimental unit, and all samples were taken from plants at that were at least 1 meter from the edges of given plots and away from gaps due to previous destructive sampling. The influence of weeds on maize growth and development were measured several times throughout each season using both nondestructive (plant height, chlorophyll) and destructive (leaf area, plant biomass) measurements. Nondestructive measurements were taken from 16 to 20 plants per plot for plant heights and from 4 plants per plot for chlorophyll measurements (from the top‐most collared leaf) (Minolta Chlorophyll Meter, SPAD‐502, Spectrum Technologies, Inc., Aurora, IL). For destructive measurements, two representative plants/rep/treatment were harvested at each time point. Plants were cut at soil level. Leaf area was obtained by stripping leaves off the stem and running them through a calibrated leaf area meter (LI‐3100C Area Meter, LI‐COR, Inc., Lincoln, NE). Plant biomass was quantified by drying plant material at 60°C until constant weight and weighing. Maize ears were harvested after physiological maturity (black layer) in the fall of each year, and grain yields were obtained and reported at 15.5% moisture. ANOVA in SAS was used to analyze the above data. Means of treatments differed when *p* < .1 for the *F* test.

### 2.2 Plant material for RNAseq analysis

Plant material for construction of sequencing libraries was harvested from plots described above on July 3, 2007, and July 2, 2008, when plants were at the V8 stage of growth in weed‐free control plots. The WR4 and WR8 treatments were harvested on the same date as the weed‐free control treatment but were developmentally lagging. Each sample consisted of pooled material from the distal 8 cm of the top‐most leaf from four field‐grown plants harvested directly into liquid nitrogen. As noted in previous similar studies, this tissue was chosen because it was unlikely to have experienced direct shading from any weeds present and was the primary source material for photosynthates needed for plant growth and also a sink material for many soil nutrients (Horvath et al., 2006). Additionally, previous transcriptome work by our group had indicated significant differences in gene expression in similar tissues following weed pressure relative to the weed‐free control treatments (Horvath et al., 2006; Moriles et al., 2012) indicating that leaf tip tissue was suitable for these analyses. Plant material from three of the four plots per treatment was collected, generating three biological replicates from each treatment/year. One sample from the WR4 2008 was lost during library preparation. Plant material was collected during the day between 11:00 and 14:00 for all treatments to avoid differences due to circadian responses.

### 2.3 Library prep and sequencing

About 1 g of leaf tissue from each plot sample (consisting of multiple leaf tips) was homogenized in liquid N and finely ground to a talc‐like powder in a precooled, porcelain mortar, and pestle. Total RNA was extracted using Trizol reagent following the manufacturer's protocol. Poly A+ RNA was extracted and the resulting mRNA was used to create RNA sequencing libraries using either the TruSeq kit (Illumina, Madison, WI) or the NEBnext Ultra Directional RNA library Prep Kit (New England Biolabs Inc., Ipswich MA). Library quality was assessed using an Agilent Bioanalyzer and quantified for pooling by qRT–PCR using the PhiX Control Kit v2 (Illumina, Madison, WI) according to manufacturer specifications. Libraries were paired‐end sequenced or single‐end sequenced on an Illumina HiSeq2000 for 100 base reads per end. Raw data and expression analysis are available from the gene expression omnibus (Accession # GSE83411).

### 2.4 Sequence analysis

There was sufficient biological replication to analyze each year's data as a separate experiment. Additionally, there was considerable variation in growth between years, suggesting large variation between years in overall gene expression patterns which would hinder false discovery statistics if both years’ data were combined. Thus, 2007 and 2008 data were each separately assessed as described below. Raw reads were quality trimmed using the program Sickle‐Quality‐Base‐Trimming (Joshi & Fass, 2011) in the iPlant discovery environment (Oliver, Lenards, Barthelson, Merchant, & McKay, 2013) using parameters of 20 for minimum quality score and 70 bases for minimum length.

Additionally, the Tuxedo suite of programs (Trapnell et al., 2012) was used to map the trimmed reads to the Tophat2‐SE programs in the iPlant discovery environment and to map the reads to the *Zea mays* Ensemble 19 annotated database in iPlant. The single‐end (SE) program was used because several of the libraries from 2008 were only sequenced as single‐end reads, so the forward read of all files were used to avoid sample bias. The resulting Binary Alignment Map (BAM) files were used to determine relative gene expression based on the fragments per kilobase per million (FPKM) as output in the gene FPKM tracking output files from the program Cufflinks2. Differential expression statistics were obtained using the Tophat2‐generated BAM files in the Cuffdiff2 program in the iPlant discovery environment. Genes were considered expressed (good) only if they had an FPKM >5 in all replicates of at least one treatment group (indicated as “good” or “bad” column labeled “>5” in Appendix S1). Genes were only considered as differentially expressed if they had *q*‐values <0.05 in both years and the change in expression was in the same direction in both years.

De novo assembly of the transcriptome was also performed using the program Trinity (Robertson et al., 2011) in the iPlant discovery environment. Open‐reading frames were identified using the program transcript decoder 1.0 in the iPlant discovery environment. Long noncoding RNAs (lncRNAs) were selected based on the criteria that the transcript had no open‐reading frames in any closely related contig (those clustered by the Trinity assembly program), was at least 300 bases long, was significantly differentially expressed (false discovery statistics <0.05) between treatments in both years based on the output from the RSEM program (Li & Dewey, 2011) that mapped reads back to the de novo assembled transcriptome, was expressed at greater than 10 transcripts per million, and had no significant homology to known maize genes.

Gene set enrichment analyses (GSEA) and subnetwork enrichment analyses (SNEA) were performed using the program Pathway studio 9.0 (Bogner et al., 2011) on all “expressed” (see above) genes based on normalized FPKM obtained from the Cufflinks output file. GSEA and SNEA were also subsequently run on subsets of all upregulated genes or all downregulated genes and also on just those genes that were significantly differentially expressed (*p* > .05) within the Pathway studio program (Appendix S3a–f: ALL, Up in treatment, or Just Significant). Arabidopsis gene annotations (based on BlastX [basic local alignment search tool] of the *Zea mays* Ensemble 19 coding sequence fasta file against the TAIR [The Arabidopsis Information Resource] 10 protein database) were used for all functional ontology attributions in the GSEA and SNEA.

## 3 RESULTS

### 3.1 Field data demonstrating weed impact

Maize height, biomass, chlorophyll content at V8, and yield at seasons end (harvest) were measured (Table 3). When measured at the V8 stage following weed removal at V4, differences in biomass, chlorophyll content (and leaf area but only in 2008), were evident but not always significantly different from weed‐free controls. Likewise, yield at the end of the season was significantly different when weeds were present only through V4 in 2008, but not in 2007. Despite greater height, leaf area, and biomass in 2007 compared to 2008 at V8, yields at the end of season were similar within treatments between these years.

**Table 3:** Plant height, leaf area, biomass, chlorophyll index at V8 and grain yield at harvest for 2007 and 2008. Letters following values indicate significance at *p* < .1. WR4 signifies data from treatments where weeds were removed at V4, and WR8 signifies data from treatments where weeds were removed at V8

Treatment	2007					2008				
	V8				Harvest	V8				Harvest
	Height, cm	Leaf area, cm^2^/plant	Biomass, g/plant	Chlorophyll, spad units	Yield, kg/ha	Height, cm	Leaf area, cm^2^/plant	Biomass, g/plant	Chlorophyll, spad units	Yield, kg/ha
Weed free	99.3a	5,161a	50.6a	50.2a	11,728a	121a	1,750a	18.7a	48.3a	12,481a
WR4	94.7b	4,263a	49.5a	51.0a	11,227a	93b	1,451a	14.5b	44.1b	11,540b
WR8	65.0c	2,834b	20.8b	44.3b	10,097b	86b	9,70b	9.0c	38.1c	10,725b

### 3.2 Yield and growth responses indicate the CWFPs in 2007 and 2008

Even with the two different weed species used, maize exposed to weeds only through V8 were significantly different from weed‐free controls in all parameters tested including the yield at seasons end, suggesting that the V8 stage was within or past the CWFP for both 2007 and 2008 (Table 3). Likewise, because the WR4 treatment caused significant differences for yield in 2008 but not in 2007, the results suggest that the CWFP might have started prior to V4 in 2008, but later than V4 in 2007. However, for plants subjected to weed stress only through V4, significant differences in height were observed in 2007 and 2008 at V8. This suggests that although V4 may have been prior to the CWFP in 2007, the weeds still resulted in reduced height well after weed removal in both years. The CWFP for maize in this location has been reported to start as early as the V2 stage of development, depending on the year and the weed species present (Moriles et al., 2012).

### 3.3 Sequencing results and mapping of fragments

cDNA fragments (9.1 million to 37.1 million raw reads) were obtained from each sequenced library (Table 4). Trimming resulted in losses ranging from 3% to 9% of the fragments from any given sample. Mapping of the single end (left reads) by Tophat2.0 resulted in 7.6 to 31.4 million reads mapping to the *Zea mays* Ensemble 19 reference genome, with between 6.6 and 27.8 million reads mapping uniquely. The Cufflinks 2.0 program identified 35,410 annotated genes with FPKM greater than 0 in all replicates of at least one treatment from the 2007 libraries; however, only 16,337 of these had FPKM values greater than 5 in all replicates of at least one treatment. Likewise, 34,737 annotated genes were expressed in the 2008 libraries, with 14,005 expressed at 5 FPKM or greater. Of the 31,671 genes expressed in both 2007 and 2008, 12,440 were expressed at greater than 5 KPKM in both years (Appendix S1).

**Table 4:** Summary of RNAseq results of maize plants sampled at V8 from the weed‐free control (Control), weeds removed at V4 (WR4), and weeds removed at V8 (WR8) treatments in 2007 and 2008

Year	Weed	Treatment	Raw reads	Trimmed reads	% Mapped	# Mapped uniquely
2007	Velvetleaf	Control	24,206,291	23,408,478	84	17,505,022
			15,373,738	14,967,801	84	11,311,049
			12,871,548	12,509,325	89	9,948,686
		WR4	22,720,273	22,055,772	85	16,504,365
			17,348,145	16,835,951	84	12,788,062
			21,466,693	20,818,818	85	15,889,868
		WR8	9,051,642	8,800,008	86	6,603,625
			37,060,647	35,974,228	87	27,531,463
			20,788,256	20,211,567	85	15,587,838
2008	Canola	Control	19,886,038	19,369,257	89	14,752,769
			16,933,675	16,519,440	89	12,918,911
			36,325,778	35,241,151	89	27,824,147
		WR4	16,636,256	15,156,777	84	11,273,466
			18,011,690	16,404,218	85	12,474,823
		WR8	14,305,066	13,058,530	89	6,713,352
			16,786,595	15,420,848	85	11,664,771
			18,492,391	16,848,573	85	12,845,495

### 3.4 Differential gene expression results

We identified 524 genes that were adequately and differentially expressed (FPKM > 5 in all replicates of at least one treatment, and *q* < 0.05) between weed‐free control treatments and WR8 treatments in 2007, and 1,315 genes in 2008 (Table 5 and Appendix S1). Of these, only 25 were differentially expressed with the same expression trend (19 were downregulated and 6 were upregulated in the weedy treatments) in both years (Table 6). Only one transcription factor encoding gene (GRMZM5G821755, a homeodomain transcription factor involved in floral meristem determination) was noted, and it was downregulated in the weedy treatments. Likewise, 128 and 129 genes were differentially expressed between the weed‐free control and the WR4 treatments which were sampled in 2007 and 2008, respectively (Table 5 and Appendix S1), of which only one, a cysteine protease superfamily protein (GRMZM2G049882), was differentially expressed in both years (Table 6). A comparison of the WR8 and the WR4 treatments indicated that 392 genes were differentially expressed in 2007 and 537 were differentially expressed in 2008 (Appendix S1). Of these, only 4 were upregulated and 6 were downregulated in the WR4 treatments as compared to the WR8 treatments in both years. One abscisic acid (ABA) regulated homeobox transcription factor (GRMZM2G051305) was among the genes commonly downregulated in both years between the WR4 treatments relative to the WR8 treatments.

**Table 5:** Number of differentially expressed genes (DEG; *q* < 0.05), and distribution of up‐ or downregulated genes in maize plants sampled at V8 from the weeds removed at V4 (WR4) and weeds removed at V8 (WR8) treatments relative to weed‐free control treatments (Control)

Year	Weed	Treatment	DEG	Up	Down
2007	Velvetleaf	WR8 vs. Control	524	144	380
		WR4 vs. Control	128	7	121
2008	Canola	WR8 vs. Control	1,315	442	873
		WR4 vs. Control	129	50	79

**Table 6:** Differentially expressed genes with common up‐ or downregulation in maize plants relative to the weed‐free control at V8 (Control), from weeds removed at V4 (WR4), and weeds removed at V8 treatments (WR8). Weed pressure in maize resulted from presence or absence of velvetleaf (2007) and canola (2008)

Treatment	Ave log2 fold change 2007	Ave log2 fold change 2008	Gene ID	Gene annotation
WR8 vs. Control	1.18	1.30	GRMZM2G106344	DC1 domain‐containing protein
	1.39	0.94	GRMZM2G018018	Major Facilitator Superfamily with SPX domain
	2.27	1.40	GRMZM2G099834	Photosystem II reaction center protein C
	2.42	1.28	GRMZM2G004224	Photosystem II reaction center protein D
	1.07	2.17	GRMZM2G062156	Polyol/monosaccharide transporter 5
	1.20	0.93	GRMZM6G761998	Zinc transporter 11 precursor
	−2.03	−1.17	GRMZM2G478160	Calcium‐binding EF‐hand family protein
	−1.03	−1.31	GRMZM2G007939	Chloroplast beta‐amylase
	−1.45	−2.36	GRMZM2G048120	Eukaryotic aspartyl protease family protein
	−1.16	−2.15	GRMZM2G048161	Eukaryotic aspartyl protease family protein
	−1.48	−1.12	GRMZM2G050961	GroES‐like family protein
	−0.89	−1.06	GRMZM5G821755	Homeobox protein 31
	−1.11	−1.47	GRMZM2G473001	Phosphoenolpyruvate carboxylase 3
	−0.79	−1.41	AC217050.4_FG001	Regulator of chromosome condensation (RCC1) family protein
	−1.77	−1.50	GRMZM2G158394	Ribonuclease T2 family protein
	−1.18	−0.86	GRMZM2G076263	Ribosomal protein S21 family protein
	−0.89	−1.32	GRMZM2G154223	Serine‐rich protein‐related
	−0.93	−0.98	GRMZM2G436710	Tetratricopeptide repeat (TPR)‐like superfamily protein
	−1.07	−2.02	GRMZM2G027447	Tonoplast intrinsic protein 2;3
	−0.95	−2.19	GRMZM2G058081	Unknown
	−0.97	−1.09	GRMZM2G085777	Unknown
	−1.77	−2.21	GRMZM2G134264	Unknown
	−1.00	−2.01	GRMZM2G342401	Unknown
	−1.10	−1.55	GRMZM2G350693	Unknown
	−1.16	−1.63	GRMZM5G839640	Unknown
WR4 vs. Control	−1.50	−0.92	GRMZM2G049882	Cysteine protease superfamily protein

### 3.5 Identification of long noncoding RNAs

De novo assembly identified 1,863 transcripts representing 525 genes which were differentially expressed in both 2007 and 2008. Of these transcripts, 161 had no long open‐reading frames (Appendix S2), but only two of these had no similarity to previously characterized maize coding sequences. Thus, the bulk of the noncoding RNAs likely represent mutations or splice variants that alter the reading frame of known maize coding sequences. Contigs (comp76348_c0_seq1 and comp157611_c0_seq1) were significantly downregulated in WR8 treatments in both 2007 and 2008, but were unchanged in WR4 treatments in 2007 or were downregulated in similar treatments in 2008.

### 3.6 Gene Set and subnetwork enrichment analysis

Forty‐two ontologies were over‐represented among genes upregulated in WR8 treatments in both 2007 and 2008 (Tables 7 and 8, and Appendix S3). Fifteen of these ontologies were associated with biotic stress responses. Seven were indicative of hormone responses with a majority implicating auxin, abscisic acid (ABA), gibberellic acid (GA), and ethylene; additionally, several hormone‐related ontologies, such as 3 jasmonic acid (JA) and 3 salicylic acid (SA) ontologies, are categorized under biotic defense. Three were indicative of nutrient or water deprivation, and one was associated with phytochrome signaling. Likewise, 47 ontologies were consistently over‐represented among genes that are downregulated when weeds were present through V8. Of these ontologies, fifteen were associated with photosynthesis and carbon metabolism, ten were associated with nitrogen responses or amino acid biosynthesis and protein production, five were associated with growth and development, and three were associated with oxidative stress. “Downstream neighbors of gibberellin” and “1‐aminocyclopropane‐1‐carboxylate synthase activity” (an enzyme involved in ethylene production) were the only hormone‐related ontologies that were identified as over‐represented among the genes downregulated in WR8 treatments.

**Table 7:** Number of over‐represented ontologies categorized, and common for up‐ or downregulated genes in 2007 and 2008, in maize plants at V8 from weeds removed at V4 (WR4) and weeds removed at V8 (WR8) treatments relative to weed‐free control treatments (Control). Weed pressure in maize resulted from presence or absence of velvetleaf (2007) and canola (2008) relative to weed‐free control treatments. The ontologies and associated genes under each category are noted below in Table 8

Category	WR8 vs. Control		WR4 vs. Control	
	Up	Down	Up	Down
Biotic defense	15	0	2	7
Flavonoids	1	0	1	3
Growth and development	4	5	1	11
Hormones	7	2	0	8
Nitrogen/amino acid/protein	0	10	2	6
Osmotic/cold	3	1	1	8
Oxidative stress	2	3	2	6
Photosynthesis/carbon	1	15	0	1
Light signaling (phytochrome)	1	0	1	0
Propanoid/lignin	0	0	0	6
Others	8	11	8	17
Total	42	47	18	73

**Table 8:** List of statistically over‐represented ontologies that were “common” between 2007 and 2008 among all up‐ or all downregulated genes (not just significantly up or down) relative to the weed‐free control from the designated conditions (WR8—weeds allowed to remain until harvest at V8; WR4—weeds removed at V4 prior to harvest at V8). Ontologies in bold and italics were common in both treatments (WR8 and WR4) and represent processes that persist even after weed removal at least until V8. Colors correspond to the arbitrary groupings of processes noted in Table 7

Common up in WR8	Common down in WR8
*Defense response*	*Response to cold*
Defense response to bacterium	*Heme binding*
Defense response to fungus	Peroxidase activity
Detection of biotic stimulus	Proteins/Chemicals Regulating Cell Processes of oxidative stress
Jasmonic acid mediated signaling pathway	Aromatic amino acid family biosynthetic process
Jasmonic Acid Signaling	Binding Partners of ribosome
Plant‐type hypersensitive response	Binding Partners of translocon
Regulation of hydrogen peroxide metabolic process	Binding Partners of tRNA
Regulation of plant‐type hypersensitive response	Downstream Neighbors of nitrate
Response to bacterium	*Nitrate transport*
Response to chitin	Proline transport
Response to jasmonic acid stimulus	*Response to nitrate*
Salicylic acid biosynthetic process	rRNA binding
*Salicylic acid mediated signaling pathway*	rRNA processing
Systemic acquired resistance, salicylic acid mediated signaling pathway	Ovule development
Hyperosmotic salinity response	Plant‐type cell wall organization
Response to osmotic stress	Proteins/Chemicals Regulating Cell Processes of phototropism
Response to water deprivation	*Regulation of cell size*
Iron ion binding	*Regulation of meristem growth*
Oxidoreductase activity, acting on single donors with incorporation of molecular oxygen	Downstream Neighbors of gibberellin
Cellular response to nitrogen starvation	Carbohydrate metabolic process
Cellular response to phosphate starvation	Chloroplast
Proteins/Chemicals Regulating Cell Processes of reproduction	Chloroplast envelope
Regulation of seed germination	Chloroplast inner membrane
Chlorophyll catabolic process	Chloroplast organization
Abscisic acid mediated signaling pathway	Chloroplast relocation
Ethylene mediated signaling pathway	Chloroplast stroma
Response to abscisic acid stimulus	Plastid
Response to auxin stimulus	Plastid chromosome
Response to ethylene stimulus	Plastid translation
Response to gibberellin stimulus	Protein targeting to chloroplast
Response to karrikin	Proteins/Chemicals Regulating Cell Processes of chloroplast organization and biogenesis
*Phytochrome Signaling*	Thylakoid
Regulation of anthocyanin metabolic process	Thylakoid membrane organization
Antiporter activity	Transcription from plastid promoter
Calmodulin binding	1‐aminocyclopropane‐1‐carboxylate synthase activity
Protein targeting to membrane	*Apoplast*
Response to hypoxia	Calcium‐mediated signaling
Sequence‐specific DNA binding	Coenzyme binding
Sequence‐specific DNA binding transcription factor activity	Downstream Neighbors of lipoic acid
Signal transduction	Iron–sulfur cluster assembly
Transcription, DNA‐dependent	Isopentenyl diphosphate biosynthetic process, mevalonate‐independent pathway
	Methyl indole‐3‐acetate esterase activity
	ncRNA metabolic process
	Nucleoid
	Positive regulation of transcription, DNA‐dependent
	Transmembrane receptor protein tyrosine kinase signaling pathway

**Common up in WR4**	**Common down in WR4**
*Defense response*	Jasmonic acid mediated signaling pathway
*Salicylic acid mediated signaling pathway*	Regulation of plant‐type hypersensitive response
Proteins/Chemicals Regulating Cell Processes of cold acclimation	Response to chitin
Monooxygenase activity	Response to fungus
Oxygen binding	Response to jasmonic acid stimulus
Proteins/Chemicals Regulating Cell Processes of organ formation	Response to wounding
*Phytochrome Signaling*	Systemic acquired resistance
Positive regulation of flavonoid biosynthetic process	Downstream Neighbors of DREB1A
Cysteine‐type endopeptidase activity	Hyperosmotic response
Cysteine‐type peptidase activity	Hyperosmotic salinity response
Integral to membrane	*Response to cold*
Kinase activity	Response to salt stress
Plant‐type vacuole membrane	Response to water deprivation
Plasma membrane	Water transport
Protein serine–threonine kinase activity	Response to temperature stimulus
Regulation of stomatal movement	Downstream Neighbors of peroxidases
Transmembrane receptor protein tyrosine kinase	*Heme binding*
Transporter activity	Oxidoreductase activity
	Oxygen binding
	Regulation of hydrogen peroxide metabolic process
	Response to oxidative stress
	Cysteine biosynthetic process
	Nitrate assimilation
	*Nitrate transport*
	Proteins/Chemicals Regulating Cell Processes of nitrate assimilation
	Proteins/Chemicals Regulating Cell Processes of nitrogen metabolism
	*Response to nitrate*
	Cell tip growth
	Cell wall
	Cell wall organization
	miRNA Targets of MIR172A
	Multidimensional cell growth
	*Regulation of cell size*
	*Regulation of meristem growth*
	Regulation of photomorphogenesis
	Root hair elongation
	Secondary cell wall biogenesis
	Negative regulation of seed germination
	Anthocyanin accumulation in tissues in response to UV light
	Flavonoid biosynthetic process
	Positive regulation of flavonoid biosynthetic process
	Auxin polar transport
	Gibberellic acid mediated signaling pathway
	Response to abscisic acid stimulus
	Response to auxin stimulus
	Response to gibberellin stimulus
	Response to karrikin
	Red or far‐red light signaling pathway
	Regulation of hormone levels
	Downstream Neighbors of HY5
	Coumarin biosynthetic process
	Lignin biosynthetic process
	Phenylpropanoid biosynthetic process
	Proteins/Chemicals Regulating Cell Processes of lignification
	Proteins/Chemicals Regulating Cell Processes of lignin biosynthesis trait
	Proteins/Chemicals Regulating Cell Processes of phenylpropanoid metabolism
	Anchored to membrane
	*Apoplast*
	Downstream Neighbors of EGTA
	Electron carrier activity
	Extracellular region
	Golgi organization
	Lipid binding
	Oligopeptide transport
	Pattern specification process
	Plasma membrane
	Polysaccharide biosynthetic process
	Protein disulfide isomerase activity
	Protein targeting to membrane
	Proteins/Chemicals Regulating Cell Processes of grain filling
	Response to mechanical stimulus
	Response to UV‐B
	UDP–glycosyltransferase activity

There were 18 and 73 ontologies identified as over‐represented among genes upregulated and downregulated (respectively) when weed‐free control treatments were compared to WR4 treatments in both 2007 and 2008 (Tables 7 and 8, and Appendix S3). Of the 18 ontologies associated with genes upregulated in the WR4 treatments, only two were associated with defense responses and one (phytochrome signaling) was associated with light signaling. Conversely, seven ontologies associated with biotic stress defense and seven associated with osmotic or cold stress were noted among genes upregulated in the WR4 treatments. However, six ontologies were associated with nitrogen responses, six associated with propanoid or lignin production, and eleven associated with growth and development processes. Unlike when weeds were present through V8, auxin‐, GA‐, and JA‐associated ontologies were over‐represented among genes downregulated in the plants from WR4 treatments. Likewise, the photosynthesis group which was downregulated when weeds remained through V8 were not different from weed‐free controls at V8 if weeds were removed at V4 (Table 8).

Ontologies that were similarly over‐represented in up‐ or downregulated genes at V8 regardless of whether the weeds were removed at V4 or allowed to remain to V8 included two defense response‐associated ontologies (defense response and SA‐mediated signaling pathway) and one light‐associated ontology (phytochrome signaling) that were associated with upregulated genes, and seven ontologies (response to cold, heme binding, nitrate transport, response to nitrate, regulation of cell size, regulation of meristem growth, and apoplast) that were over‐represented among downregulated genes (Table 8).

## 4 DISCUSSION

### 4.1 Despite large differences between years, weeds consistently altered growth responses and gene expression of maize

At V8, there were large growth and biomass differences observed in all treatments between years (Table 3), which are difficult to explain based on observations of crop health or growing conditions. However, when present through V8, weeds significantly impacted all measured growth and development parameters by the V8 stage of growth in both years. Comparisons between years identified very few genes that were commonly induced or repressed by weeds in both 2007 and 2008, and even fewer genes when weeds were removed several weeks prior to sampling (Table 6; Appendix S1). This may suggest significant environmental effects, differences due to weed type or quantity, and/or a high number of false negatives in each dataset due to the stringency of the statistical significance. That said, there were a few genes that were differentially expressed consistently. This is even more remarkable given the large differences in height, leaf area, and biomass between years at the time of sampling and indicates the robust nature of these differences in response to weed presence. It should also be noted that glyphosate was used to remove weeds in both years of the study. Thus it is possible that some of the changes in gene expression could be due to glyphosate treatment rather than to the presence of weeds. However, transcriptome analysis of glyphosate‐resistant soybean treated with glyphosate showed negligible changes in gene expression (Zhu et al., 2008), and our plants were treated with glyphosate nearly a month prior to sample collections. Additionally, many of the changes we observed are characteristic to previous studies involving corn responses to weeds where other herbicides were used to control weeds (Horvath et al., 2006), or in other plant systems—including many observed in weed‐stressed teosinte that was not glyphosate‐resistant (S. Bruggeman, unpublished data). Thus, although it cannot be ruled out, it is unlikely that many of changes we observed here are due to the glyphosate treatment.

### 4.2 Differentially expressed genes indicate a possible role for photosynthetic, oxidative stress, transport processes, growth, and nitrogen use during weed stress

Weed presence through V8 consistently resulted in upregulation of two chloroplast encoded photosystem II reaction center protein genes (GRMZM2G099834 and GRMZM2G004224). Photosystem II reaction center proteins are involved in development of the photosynthetic apparatus, and the increased abundance of transcripts encoding these two proteins suggests that weeds might have divergent effects on photosynthesis since, as noted below, many photosynthesis‐related processes are downregulated by constant weed presence through V8. Indeed, previous studies confirmed consistent downregulation of several other photosystem II genes in weed‐stressed maize (Moriles et al., 2012) and in downregulation of photosynthetic processes in general that were observed during weed stress at V12 (Horvath et al., 2006). Additionally, recent work has implicated weed‐induced oxidative stress as a mechanism for photosystem II damage (C.J. Swanton, personal communication). Such damage might require higher expression of the protein PS IID, which we found to be consistently upregulated by weed presence.

The fact that no genes were consistently upregulated relative to weed‐free controls is consistent with the relatively few genes differentially expressed between weed‐free control treatments and WR4 treatments at V8. This could indicate that early weed presence has little impact on gene expression. However, this seems unlikely given the number of gene expression differences observed at V4 in earlier microarray studies (Moriles, 2011), which indicated that photosynthesis was downregulated at V4 in the presence of weeds. This might also suggest that only a few changes in gene expression persisted through V8 once weeds were removed. This is of interest since weeds, when present during the CWFP, have a profound impact on crop development even if they are subsequently controlled later in the growing season (Zimdahl, 1988).

Genes that are downregulated when weeds were present through V8, relative to weed‐free controls (Table 6), highlighted several involved in photosynthesis and carbon metabolism including genes encoding proteins with similarity to a chloroplast beta‐amylase (GRMZM2G007939), and phosphoenolpyruvate carboxylase 3 (GRMZM2G473001). In addition to these, two different eukaryotic aspartyl protease family protein coding genes (GRMZM2G048161 and GRMZM2G048120), and genes encoding a ribosomal protein S21 family protein (GRMZM2G076263), and a tonoplast intrinsic protein 2;3 (GRMZM2G027447) possibly involved in ammonium transmembrane transport indicate that weeds impact nitrogen signaling and protein production and degradation. In earlier studies, both weeds and low nitrogen levels in the soils resulted in similar changes in gene expression—particularly in regard to photosynthetic gene expression and carbon metabolism (Moriles et al., 2012). These observations are also consistent with the observation that carbon and nitrogen levels can interact with redox states and result in altered expression of genes required for the photosynthetic apparatus (Paul & Foyer, 2001).

Several other genes of interest include one that encodes a regulator of chromosome condensation family protein (*RCC1*). Given that weed presence during the CWFP impacts plant development (even if subsequently removed), it has been hypothesized (Horvath et al., 2006) that some epigenetic markers might be altered by weed presence during this critical developmental window. Thus, the differential expression of a gene potentially involved in altering chromatin condensation is of considerable interest. Likewise, we observed the downregulation of a homologue of HOMEOBOX PROTEIN 31 (GRMZM5G821755), which encodes a critical developmentally active transcription factor involved in regulating floral development and is also downregulated to a large extent by stress responses and SA in Arabidopsis (TAIR.arabidopsis.org and links therein). This gene is also of interest since one of the mechanisms through which weeds might impact yield involves early induction of flowering via shade avoidance signals (Cerdan & Chory, 2003). Only one gene was consistently downregulated at V8 relative to the weed‐free controls when weeds were removed previously at V4. This gene encodes a putative cysteine protease that, in Arabidopsis, is also primarily induced during floral development. It is unclear why it might be downregulated at V8 if weeds were present to V4.

### 4.3 Gene set and subnetwork enrichment analyses indicate that weeds induce specific defense and hormone responses and inhibit photosynthesis, growth, and development

Because of the strict criteria used for defining differential expression, it is likely that a large number of false negatives were present in the differentially expressed gene dataset. GSEA and SNEA overcome this issue to some extent because different genes from either year of treatment can implicate the same pathway or process. Gene expression as determined by the Cuffdiff2.0 program, along with functional data derived from similarity of maize genes to arabidopsis, was used to generate lists of over‐represented ontologies. These data corroborate observed downregulation of photosynthetic processes observed in the small number of differentially expressed genes (Tables 7 and 8) and in similar studies (Horvath et al., 2006; Moriles et al., 2012). They are also consistent with the downregulation of observed chlorophyll content observed when weeds were present through V8. Interestingly though, the expression of these genes did not appear to be repressed at V8 if the weeds were removed by V4. This indicates that downregulation of the photosynthetic processes may not be repressed early in the CWFP (which would be contradictory to previous studies; Moriles, 2011), or if they are, may not be maintained following weed removal. However, microarray studies conducted on plants collected at V4 from 2008 indicates that photosynthetic processes were repressed by weeds when present through V4 (Moriles, 2011).

Likewise, the altered nitrogen signaling observed from the limited number of consistently differentially expressed genes was strengthened by the GSEA results. Indeed, when GSEA and SNEA were run only on the significantly differentially expressed genes from WR8 treatments, the vast majority of over‐represented ontologies indicated in both years were heavily populated by nitrogen responses and photosynthesis. Additionally, membrane and lipid processes were also noted to be downregulated (Table 8 and Appendix S3) suggesting possible impacts on cellular growth.

Various hormone‐associated ontologies were over‐represented among upregulated genes in response to weed presence through V8. These included several linked to auxin, GA, ABA, and ethylene. JA and SA were also implicated among biotic stress response‐associated ontologies. Previous work has implicated shade avoidance responses as likely mechanisms for weed‐induced crop losses (Liu et al., 2009). Consistent with this are the observed alterations in phytochrome and auxin signaling that were previously associated with shade‐induced reduced branching and other shade avoidance‐linked developmental processes (Finlayson, Krishnareddy, Kebrom, & Casal, 2010). Likewise, altered GA/ABA signaling was also previously associated with shade avoidance (Leivar & Quail, 2011). Interestingly, most of the indicated hormone responses, with the exception the SA, were over‐represented among downregulated genes at V8 if the weeds had been removed at V4. This may indicate a compensatory or rebound effect on these signaling processes if weeds are removed early in the CWFP.

Because weed presence early in the CWFP can impact season‐long growth and development, it was of considerable interest to identify processes that were altered at V8 in both the WR8 and WR4 treatments as these processes might be those involved in long‐term alteration of crop growth. We observed a significant impact of weed presence on grain yield (14%) in the treatments where weeds were removed at V4 in 2008 and nearly a 5% loss in 2007. Additionally, maize perceived and responds to weeds very early in the growing season—perhaps even before emergence (Mckenzie‐Gopsill, Lee, Lukens, & Swanton, 2016). SA and defense responses observed at V8, even after weed removal at V4, suggests that persistent induction of defenses might be the cause of season‐long yield losses. Although decreased ratios of red to far‐red light‐associated with weed presence have been shown to reduce SA levels in several studies (Izaguirre, Mazza, Biondini, Baldwin, & Ballaré, 2006; de Wit et al., 2013), upregulated defense responses have been implicated in weed stress (Cipollini, 2005; Faigón‐Soverna et al., 2006; Subrahmaniam et al., 2018). Interspecies competition between corn and soybean also resulted in increased production of SA in corn roots (Gao et al., 2014). The upregulation of SA‐associated responses we observed in our GSEA could be due to overlap in genes induced by both SA and oxidative stress or defense rather than to SA per se.

SA plays a role in the hypersensitive response of plants to pathogens and the subsequent oxidative burst response of impacted cells (Torres, Jones, & Dangl, 2006). At least one study has shown that blocking the oxidative stress response of plants can negate many of the responses of plants to weeds (Afifi & Swanton, 2012). Additionally, hyperstimulation of SA has been associated with reduced plant growth (Rivas‐San Vicente & Plasencia, 2011). Thus, the over‐representation of genes associated with cell size and meristem growth among downregulated genes could be linked to the indicated induction of the plant defense response through SA or oxidative stress signaling. Our observations here and previous work on crop response to weeds or enhanced far‐red light strongly implicate cross‐talk between SA/biotic defense signaling and weed‐induced responses of maize (Afifi et al., 2015; Horvath et al., 2006; Mckenzie‐Gopsill et al., 2016; de Wit et al., 2013). Altering SA signaling responses or downstream oxidative stress responses might allow manipulation and possible repression of the response of maize to weeds. Such weed‐tolerant maize would allow greater flexibility in the timing of herbicide application and/or provide novel intercropping/cover‐cropping opportunities for growers.

One surprising observation was the preferential upregulation of genes involved in nutrient and water uptake—specifically those with ontologies associated with cellular response to nitrogen and phosphate starvation and with water deprivation and osmotic stress in WR8 treatments (Appendix S3g). However, recently, there have been several studies that implicate ABA signaling, which is also a notable signal during water stress, with shade avoidance responses (Yang & Li, 2017). Additionally, one of the few genes consistently induced when maize was grown in the presence of weeds through V8 encodes a zinc transporter protein. The zinc transporter is similar to *ZIP1* of Arabidopsis and is induced by low zinc levels in several plant species (Van de Mortel et al., 2006). Although these observations might have been expected if weeds were reducing the levels of nutrients or water in the soil, loss of soil nutrients was not observed in similar studies (Horvath et al., 2006; Moriles et al., 2012). Additionally, given the supplementation of these resources in the field plots, it was not expected that these resources would be limiting. Indeed, previous studies have provided evidence that resource limitation is not the primary reason why weeds inhibit crop yield (Afifi et al., 2015; Liu et al., 2009; Page et al., 2012). Our data suggest that even in high input agricultural setting crops can sense and respond to weed presence by inducing systems involved in dealing with resource limitation; perhaps, even when they are not needed.

The other observation of interest is the significant over representation in downregulated genes of ontologies associated with nitrogen transport and use in both WR8 and WR4 treatments. This suggests that even though weeds were removed early in the CWFP, the ability of the plant to take up and use nitrogen was impaired at least to V8. This may explain why genes associated with nitrogen starvation are generally upregulated in response to weeds. This observation is consistent with earlier studies that suggested many of the changes in gene expression resulting from weed stress were also observed in nitrogen‐starved plants (Moriles et al., 2012). It should be emphasized here that the plants at V8 were not likely to be starved for nitrogen based on earlier analyses of soil nitrogen levels in the same fields under similar nitrogen application strategies (Moriles et al., 2012).

### 4.4 Differentially expressed lncRNAs

Some lncRNAs have been shown to have regulatory roles in expression of genes involved in plant development (Zhang & Chen, 2013). Thus, the indication that several lncRNAs were differentially expressed at reasonably high levels in response to weed pressure is of interest. Two in particular (comp157611_c0_seq1 and comp76348_c0_seq1; Appendix S1) had very similar expression patterns and were both downregulated in WR8 treatments. Both have good levels of expression in weed‐free control treatments with an average FPKM between 25 and 29. Both have similarity to RNAs that were previously cloned and sequenced in maize and which were present in the Phytozome v11 database (phytozome.jgi.doe.gov/pz/portal.html). Neither has similarity to any other sequences in the nonredundant database besides the single hit for each from maize sources, and both have reasonably complex nucleotide sequence structure. Comp157611_c0_seq1 has a close match to sequences located on chromosome 7:81098719..81099439, and comp76348_c0_seq1 is related to sequences located on chromosome 2:103844268..103845265. No function has been assigned to these loci; however, the consistent differential expression in response to weed presence suggests that they are regulated by factors responsive to weed pressure. Further analyses of these lncRNAs seem warranted.

## 5 CONCLUSION

We investigated differences in gene expression in maize (i) growing under field conditions in response to weed pressure through the V8 stage of development relative to weed‐free controls and, (ii) to maize that experienced weed pressure during the early CWFP (through the V4 stage of development) that were manifested at V8. We identified a small set of genes that were consistently differentially expressed in two different years with different weed species. Gene expression data and subsequent gene set and subnetwork enrichment analyses provide evidence for physiological differences associated with altered photosynthetic processes, hormone signaling, altered nitrogen use and transport, and biotic stress responses. This work has also provided several possible targets, such as SA/plant defense signals and a small number of developmentally important genes, for manipulating the response of maize to weeds. Finally, observed differences in the expression of lncRNAs in the maize response to weeds are intriguing, and opens novel avenues for investigation into the function of these transcription units. Considerable work is needed to test the various hypotheses that have been and are yet to be developed from this dataset.

## AUTHORS CONTRIBUTIONS

Dr.s Horvath, Clay, and Bruggeman conceived of and designed the experiments. They also collected and prepared materials along with Janet Moriles‐Miller. Dr.s Horvath, Scheffler, and Anderson facilitated the sequencing. Dr.s Horvath, Bruggeman, Clay, and Dogramaci analyzed the data. Dr.s Horvath, Bruggeman, Clay, Anderson, Dogramaci, and Foley assisted in writing the manuscript.

## References

[pld357-bib-0001] Afifi, M. , Lee, E. , Lukens, L. , & Swanton, C. J. (2015). Thiamethoxam as a seed treatment alters the physiological response of maize (*Zea mays*) seedlings to neighbouring weeds. Pest Management Science, 71, 505–514. 10.1002/ps.3789 24700817

[pld357-bib-0002] Afifi, M. , & Swanton, C. J. (2011). Maize seed and stem roots differ in response to neighbouring weeds. Weed Research, 51, 442–450. 10.1111/j.1365-3180.2011.00865.x

[pld357-bib-0003] Afifi, M. , & Swanton, C. J. (2012). Early physiological mechanisms of weed competition. Weed Science, 60, 542–551. 10.1614/WS-D-12-00013.1

[pld357-bib-0004] Bogner, V. , Leidel, B. A. , Kanz, K. G. , Mutschler, W. , Neugebauer, E. A. , & Biberthaler, P. (2011). Pathway analysis in microarray data: A comparison of two different pathway analysis devices in the same data set. Shock, 35, 245–251. 10.1097/SHK.0b013e3181fc904d 20926982

[pld357-bib-0005] Carlson, H. L. , & Hill, J. E. (1985). Wild oat (*Avena fatua*) competition with spring wheat: Effects of nitrogen fertilization. Weed Science, 34, 29–33.

[pld357-bib-0006] Cerdan, P. D. , & Chory, J. (2003). Regulation of flowering time by light quality. Nature, 423, 881–885. 10.1038/nature01636 12815435

[pld357-bib-0007] Cipollini, D. (2005). Interactive effects of lateral shading and jasmonic acid on morphology, phenology, seed production, and defense traits in *Arabidopsis thaliana* . International Journal of Plant Science, 166, 955–959. 10.1086/432896

[pld357-bib-0008] Clay, S. A. , Clay, D. E. , Horvath, D. P. , Pullis, J. , Carlson, C. G. , Hansen, S. , & Reicks, G. (2009). Maize response to competition: Growth alteration vs yield limiting factors. Agronomy Journal, 101, 1522–1529. 10.2134/agronj2008.0213x

[pld357-bib-0009] DiTomaso, J. M. (1995). Approaches for improving crop competitiveness through the manipulation of fertilization strategies. Weed Science, 43, 491–497.

[pld357-bib-0010] de Wit, M. , Spoel, S. H. , Sanchez‐Perez, G. F. , Gommers, C. M. M. , Pieterse, C. M. J. , Voesenek, L. A. C. J. , & Pierik, R. (2013). Perception of low red:far‐red ratio compromises both salicylic acid‐ and jasmonic acid‐dependent pathogen defenses in Arabidopsis. The Plant Journal, 75, 90–103. 10.1111/tpj.12203 23578319

[pld357-bib-0011] Faigón‐Soverna, A. , Harmon, F. G. , Storani, L. , Karayekov, E. , Staneloni, R. J. , Gassmann, W. , … Yanovsky, M. J. (2006). A constitutive shade‐avoidance mutant implicates TIR‐NBS‐LRR proteins in Arabidopsis photomorphogenic development. The Plant Cell, 18, 2919–2928. 10.1105/tpc.105.038810 17114357 PMC1693933

[pld357-bib-0012] Finlayson, S. A. , Krishnareddy, S. R. , Kebrom, T. H. , & Casal, J. (2010). Phytochrome regulation of branching in Arabidopsis. Plant Physiology, 152, 1914–1927. 10.1104/pp.109.148833 20154098 PMC2850038

[pld357-bib-0013] Gao, X. , Wu, M. , Xu, R. , Wang, X. , Pan, R. , Kim, H.‐J. , & Liao, H. (2014). Root interactions in a maize/soybean intercropping system control soybean soil‐borne disease, Red Crown Rot. PLoS ONE, 9(5), e95031 10.1371/journal.pone.0095031 24810161 PMC4014482

[pld357-bib-0014] Horvath, D. P. , Gulden, R. , & Clay, S. A. (2006). Microarray analysis of late‐season velvetleaf (*Abutilon theophrasti*) effect on maize. Weed Science, 54, 983–994. 10.1614/WS-06-103R.1

[pld357-bib-0015] Horvath, D. , Hansen, S. , Pierik, R. , Yan, C. , Clay, D. , Scheffler, B. , & Clay, S. (2015). RNAseq reveals weed‐induced PIF3‐like as a candidate target to manipulate weed stress response in soybean. New Phytologist, 207, 196–210. 10.1111/nph.13351 25711503

[pld357-bib-0017] Izaguirre, M. M. , Mazza, C. A. , Biondini, M. , Baldwin, I. T. , & Ballaré, C. L. (2006). Remote sensing of future competitors: Impacts on plant defenses. Proceedings of the National Academy of Sciences of the United States of America, 103, 7170–7174. 10.1073/pnas.0509805103 16632610 PMC1459035

[pld357-bib-0018] Joshi, N. A. , & Fass, J. N. (2011). Sickle: A sliding‐window, adaptive, quality‐based trimming tool for FastQ files (Version 1.33) [Software]. Retrieved from https://github.com/najoshi/sickle.

[pld357-bib-0019] Kim, D. , Pertea, G. , Trapnell, C. , Pimentel, H. , Kelley, R. , & Salzberg, L. (2013). TopHat2: Accurate alignment of transcriptomes in the presence of insertions, deletions and gene fusions. Genome Biology, 14, R36 10.1186/gb-2013-14-4-r36 23618408 PMC4053844

[pld357-bib-0020] Knezevic, S. Z. , Evans, S. P. , Blankenship, E. E. , Van Acker, R. C. J. , & Lindquist, L. (2002). Critical period of weed control: The concept and data analysis. Weed Science, 50, 773–786. 10.1614/0043-1745(2002)050[0773:CPFWCT]2.0.CO;2

[pld357-bib-0021] Leivar, P. , & Quail, P. H. (2011). PIFs: Pivotal components in a cellular signaling hub. Trends in Plant Science, 16, 19–28. 10.1016/j.tplants.2010.08.003 20833098 PMC3019249

[pld357-bib-0022] Li, B. , & Dewey, C. N. (2011). RSEM: Accurate transcript quantification from RNA‐Seq data with or without a reference genome. BMC Bioinformatics, 12, 323 10.1186/1471-2105-12-323 21816040 PMC3163565

[pld357-bib-0023] Liu, J. G. , Mahoney, K. J. , Sikkema, P. H. , & Swanton, C. J. (2009). The importance of light quality in crop‐weed competition. Weed Research, 49, 217–224. 10.1111/j.1365-3180.2008.00687.x

[pld357-bib-0024] Mckenzie‐Gopsill, A. G. , Lee, E. , Lukens, L. , & Swanton, C. J. (2016). Rapid and early changes in morphology and gene expression in soya bean seedlings emerging in the presence of neighbouring weeds. Weed Research, 56, 267–273. 10.1111/wre.12207

[pld357-bib-0025] Moriles, J. C. (2011). Early maize growth and development in response to weed competition, altered light quantity, and light quality. M.S. thesis. Brookings, SD: South Dakota State University. 147 p.

[pld357-bib-0026] Moriles, J. , Hansen, S. , Horvath, D. P. , Reicks, G. , Clay, D. E. , & Clay, S. A. (2012). Microarray and growth analyses identify differences and similarities of early maize response to weeds, shade, and nitrogen stress. Weed Science, 60, 158–166. 10.1614/WS-D-11-00090.1

[pld357-bib-0027] Nleya, T. , Chungu, C. , & Kleinjan, J. (2016). Maize growth and development In Clay D. E., Carlson C. G., Clay S. A., & Byamukama E. (Eds.), iGrow maize: Best management practices (Chapter 5). Brookings, SD: South Dakota State Univ. Retrieved from http://igrow.org/up/resources/03-5000-2016-05.pdf (accessed Dec. 15, 2016)

[pld357-bib-0028] Oliver, S. L. , Lenards, A. J. , Barthelson, R. A. , Merchant, N. , & McKay, S. J. (2013). Using the iPlant collaborative discovery environment. Current Protocols in Bioinformatics, 1, 22 10.1002/0471250953.bi0122s42 23749752

[pld357-bib-0029] Page, E. R. , Cerrudo, D. , Westra, P. , Loux, M. , Smith, K. , Foresman, C. , … Swanton, C. J. (2012). Why early season weed control is important in maize. Weed Science, 60, 423–430. 10.1614/WS-D-11-00183.1

[pld357-bib-0030] Page, E. R. , Tollenaar, M. , Lee, E. A. , Lukens, L. , & Swanton, C. J. (2009). Does shade avoidance contribute to the critical period for weed control in maize (*Zea mays* L.)? Weed Research, 49, 563–571. 10.1111/j.1365-3180.2009.00735.x

[pld357-bib-0031] Paul, M. J. , & Foyer, C. H. (2001). Sink regulation of photosynthesis. Journal of Experimental Botany, 52, 1383–1400. 10.1093/jexbot/52.360.1383 11457898

[pld357-bib-0032] Rivas‐San Vicente, M. , & Plasencia, J. (2011). Salicylic acid beyond defence: Its role in plant growth and development. Journal of Experimental Botany, 62, 3321–3338. 10.1093/jxb/err031 21357767

[pld357-bib-0033] Robertson, G. , Schein, J. , Chiu, R. , Corbett, R. , Field, M. , Jackman, S. D. , … Birol, I. (2011). *De novo* assembly and analysis of RNA‐seq data. Nature Methods, 7, 909–912. 10.1038/nmeth.1517 20935650

[pld357-bib-0034] Schnable, P. S. , Ware, D. , Fulton, R. S. , Stein, J. C. , Wei, F. , Pasternak, S. , … Wilson, P. (2009). The B73 maize genome: Complexity, diversity, and dynamics. Science, 326, 1112–1115. 10.1126/science.1178534 19965430

[pld357-bib-0035] Subrahmaniam, H. J. , Libourel, C. , Journet, E.‐P. , Morel, J.‐B. , Munos, S. , Niebel, A. , … Roux, F. (2018). The genetics underlying natural variation of plant–plant interactions, a beloved but forgotten member of the family of biotic interactions. The Plant Journal, 93, 747–770. 10.1111/tpj.13799 29232012

[pld357-bib-0036] Torres, M. A. , Jones, J. D. , & Dangl, J. L. (2006). Reactive oxygen species signaling in response to pathogens. Plant Physiology, 141, 373–378. 10.1104/pp.106.079467 16760490 PMC1475467

[pld357-bib-0037] Trapnell, C. , Roberts, A. , Goff, L. , Pertea, G. , Kim, D. , Kelley, D. R. , … Pachter, L. (2012). Differential gene and transcript expression analysis of RNA‐seq experiments with TopHat and Cufflinks. Nature Protocols, 7, 562–578. 10.1038/nprot.2012.016 22383036 PMC3334321

[pld357-bib-0038] Van de Mortel, J. E. , Almar‐Villanueva, L. , Schat, H. , Kwekkeboom, J. , Coughlan, S. , Moerland, P. D. , … Aarts, M. G. (2006). Large expression differences in genes for iron and zinc homeostasis, stress response, and lignin biosynthesis distinguish roots of *Arabidopsis thaliana* and the related metal hyperaccumulator *Thlaspi caerulescens* . Plant Physiology, 142, 1127–1147. 10.1104/pp.106.082073 16998091 PMC1630723

[pld357-bib-0040] Yang, C. , & Li, L. (2017). Hormonal regulation in shade avoidance. Frontiers in Plant Science, 8, 1527 10.3389/fpls.2017.01527 28928761 PMC5591575

[pld357-bib-0041] Young, F. L. , Wyse, D. L. , & Jones, R. J. (1984). Quackgrass (*Agropyron repens*) interference on maize (*Zea mays*). Weed Science, 32, 226–234.

[pld357-bib-0042] Zhang, Y. C. , & Chen, Y. Q. (2013). Long noncoding RNAs: New regulators in plant development. Biochemical and Biophysical Research Communications, 436, 111–114. 10.1016/j.bbrc.2013.05.086 23726911

[pld357-bib-0043] Zhu, J. , Patzoldt, W. L. , Shealy, R. T. , Vodkin, L. O. , Clough, S. J. , & Tranel, P. J. (2008). Transcriptome response to glyphosate in sensitive and resistant soybean. Journal of Agriculture and Food Chemistry, 56, 6355–6363. 10.1021/jf801254e 18636734

[pld357-bib-0044] Zhu, J. , Vos, J. , Van der Werf, W. , Van der Putten, P. E. L. , & Evers, J. B. (2014). Early competition shapes maize whole‐plant development in mixed stands. Journal of Experimental Botany, 65, 641–653. 10.1093/jxb/ert408 24307719 PMC3904716

[pld357-bib-0045] Zimdahl, R. L. (1988). The concept and application of the critical weed‐free period In Altieri M. A., & Liebman M. (Eds.), Weed management in agroecosystems: Ecological approaches (pp. 145–155). Boca Raton, FL: CRC Press.

[pld357-bib-0046] Zimdahl, R. L. (2007). Weed‐crop competition: A review. Ames, IA: Blackwell Publishing.

