# Supplementary material for: Weed presence altered biotic stress and light signaling in maize even when weeds were removed early in the critical weed‐free period
Source: Plant Direct. 2018 Apr 23;2(4):e00057. doi: 10.1002/pld3.57 (PMC6508562; doi:10.1002/pld3.57)
Supplement: Supplementary file 4 [file PLD3-2-e00057-s004.docx]

**Supplemental Materials**

Supplemental file 1: Excel spread sheets for 2007 and 2008 data showing normalized expression data for all three replicates for all maize genes in the Ensemble 19 reference genome along with associated annotations, acceptable expression values (FPKM >5 for all replications of any given treatment), and pairwise significance (p values and q-values) of the three treatments (weed-free control, WR8, and WR4) as generated from the CuffDiff2 program.

Supplemental file 2: Excel spreadsheet showing expression data and annotations from the genes that were determined to be differentially expressed from the de novo assembly of the transcriptomic data as determined by the programs Trinity and RSEM.

Supplemental file 3: Excel spread sheets showing raw data for gene set and subnetwork enrichment analysis for each pairwise comparison (weedy through V8 vs weed-free control (**WR8 vs C**), weed removed at V4 vs weed-free control (**WR4 vs C**), and weeds removed vs weedy (**WR4 vs WR8**) for each of the two years (2007 and 2008). Analyses were run on the entire dataset composed of all expression data for all “good” genes (**All**) or on data from “good genes that were: on average upregulated when weed remained through V8 (**Up in WR8**), on average upregulated in weed-free controls at V8 (**Up in Controls**), or on average upregulated at V8 when weeds were removed at V4 (**Up in WR4**), or where just those “good” genes that were significantly expressed (p-value <0.05) (**Just significant**).
