# Supplementary material for: Weed presence altered biotic stress and light signaling in maize even when weeds were removed early in the critical weed‐free period
Source: Plant Direct. 2018 Apr 23;2(4):e00057. doi: 10.1002/pld3.57 (PMC6508562; doi:10.1002/pld3.57)
Supplement: Supplementary file 5 [file PLD3-2-e00057-s005.pdf]

Response to reviewers:

RE: Weed Presence Altered Biotic Stress and Light Signaling in Maize Even When Weeds Were Removed Early in the Critical Weed Free Period

Dear Dr. Horvath:

Thank you for submitting to Plant Direct. All required reviews have been returned and we have now finished our evaluation of your manuscript. In light of the reviewers' and editor's comments, further minor revisions revisions are needed before the paper can be accepted for publication in Plant Direct. Most of the comments should be taken as suggestions for improvement, but are not required changes. However, a few of the points reviewer #2 highlights are (confirming the appropriate table/gene list for GSEA analysis and missing supplemental files and details of the experiments) are needed to confirm to our standards for methods being clearly defined and understandable. We don't anticipate having to send this manuscript out for further review.

Please view the editors' and reviewers' comments below and use their suggestions as a guide while you work on your revision.

When uploading the revised version of this article, please be sure to include the following:

-A word document that contains your response to the reviewers. You should respond to each reviewer comment and note the changes made to the manuscript. If you do not agree with a reviewer's comment and choose not to make a suggested revision, please explain why. Please try to provide as complete an answer as possible to each reviewer's criticisms.
-A tracked changes document with each change highlighted
- A clean version of the latest version of the manuscript

The reviewers and I think the manuscript is sound and well written. We are only requesting some revisions that address the suggestions made by reviewer #2. They do not involve extra experimentation and we think they will improve the manuscript.

To upload your revision, please click the link below.
https://plantdirect.msubmit.net/cgi-bin/main.plex?el=A5Lr2DL6A6FZY6I6A9ftdkQGbOFZPRR7pfm62wghgZ

In order to provide as timely a service as possible, we ask that your revision is resubmitted within three months after receipt of this request. If an extension is needed, please send a request, along with a brief explanation, to the editorial office at plantdirect@wiley.com .

Thank you very much for giving us an opportunity to review your work. I look forward to receiving the next version.

Sincerely,

Ruben Rellan-Alvarez

Ivan Baxter

Editor, Plant Direct

--- Reviewer comments:

Reviewer #1 :

This study takes on the major enigma of the yield loss due to early weed competition in maize and does describe some gene expression differences but fails to identify a clear biochemical response. The effort was well designed and well carried out. The detailed analysis of up and down regulated genes seems correct, but stops short of identifying a distinct "culprit". The discussion of comparing our findings to theirs before us gets tedious. It appears to be a complete review of the literature for each of the ontologies but enhanced understanding is not provided. This discussion section might be shortened. The authors it seems want to leave nothing out in their attention to detail, but in fact all of the comparisons do not really help. The paper will be a foundation stone as they and others continue to seek an explanation and the results here can be used to develop new hypotheses. A very nice thorough study and the comparison of weeding at W4 to W8 is very interesting.

We fully agree with the reviewers remarks. In the revision, we have focused just on those processes that appear to be altered in both the W4 and W8 vs control since such processes are likely the key to understanding what happens with weed exposure during the CWFP that are likely to impact growth and development and yield even if the weeds are removed thereafter. We somewhat disagree however that distinct culprit was not identified. The observed altered PIF responses have long been associated with shade avoidance responses proposed to have a significant role in plant competition. Also, the obvious induction of biotic stress – particularly SA signaling that is carries on even after weed removal seems to be both consistent with some past observations, and provides an explanation for the long term reduced growth and yield associated with weed stress and which provides a possible reason why weeds might result in such growth/yield losses in light of the probably minimal impact of direct competition for resources. Hopefully this is better highlighted in the revised version.

Reviewer #2 :

The manuscript from Horvath et al seeks to explain the effects of early season weed pressure on yield through changes in gene expression in maize. The study was designed specifically to compare effects of past vs present weed pressure. The authors show that even when weeds are removed early in the

CWFP, there is a yield penalty, and that weed pressure during the CWFP up to V8 has an even stronger effect on yield. Samples taken at the V8 time point were subjected to RNAseq to understand the differences between weed free and weed-stressed plants. The study fills a gap in the current literature by examining the effects of early season weed pressure while the plants are still relatively young (V8), as compared to previous studies (Horvath et al 2006, for example) that examine the effect of persistent weed pressure through later stages of growth. The interpretation of the results is thorough and some specific genes and biological processes are implicated in the observed yield reduction from early season weed pressure, even when the weeds were removed. I was particularly pleased with the discussion of the results. Over all the study was well designed and appears to have been executed in a sound manner. Below I will summarize major and minor points that could be addressed to improve the content and delivery of the manuscript.

Major Points

Lines 274-276: It is stated that only one regulatory gene was identified. I disagree with this assessment. The statement could be changed to say that "only one transcription factor..." to make it more accurate. I would argue that many of the genes in Table 6 have regulatory roles. In fact, one of them has "regulator" in its name, and is discussed in the context of regulating epigenetic modifications in the discussion (lines 413-418).

An excellent point, and we agree fully. We have revised the sentence to read : “Only one transcription factor encoding gene (GRMZM5G821755,…”

Lines 280-286: The data being discussed is not included in Tables 5 and 6. If it is worth mentioning, then it is worth including in the Tables. Please add to the Tables.

Table 5 is just showing the numbers of significantly up and down regulated genes. There are far too many to list in a table in the manuscript. However, I am guessing that the reviewer did not have access to supplemental table 1 which has the normalized expression level of all the annotated corn genes, as well as the p and q values generated by each comparison. We have further gone into this supplemental table and colored the p and q value columns of all significantly differentially expressed and up regulated (relative to the control) orange, and all those down-regulated green.

Lines 299-300: The description of the gene sets used for GSEA is lacking or confusing to me. "...genes up-regulated in WR8 treatments in both 2007 and 2008" means to me the list of genes contained in Table 6, for which it would not make sense to perform GSEA (since the number is so low). Please add a sentence or two to describe the gene sets used for GSEA. I think the gene sets used are actually listed in Table 5?

As noted in the revised methods, the gene sets included all “expressed” genes – which were then separated into those that were up-regulated or down-regulated in a given treatment relative to the control (or to WR8 in the case of the comparison of WR4 vs WR8). We also ran an analysis on only

significantly differentially regulated genes. We did not limit our enrichment analysis to just those genes noted in table 6. GSEA and SNEA are more appropriately run on the entire data set rather than limiting the analyses to just those genes that were arbitrarily assigned significance. See Subramanian et al. PNAS 2005.

Lines 298-333: This section of the results repeatedly refers to the supplemental file 3. Because of its value in interpreting the results, I suggest that the information in this file, particularly in tabs 3g, 3h, and 3i, would be better suited as a figure(s) in the actual paper.

We fully agree, and have now included this data in a new table (Table 8). We have also included the coloring scheme in Table 7 to help identify the manually grouped ontologies.

Lines 365-368: A statement is made about components of the photosynthetic apparatus being down-regulated. This statement is apparently based on the GSEA analysis presented in Table 7. However, the rest of the section deals not with GSEA, but with the differentially expressed genes listed in Table 6. I suggest moving the content of lines 365-368 to the beginning of the next discussion section dealing with GSEA. It seems out of place in its current location.

The information in that opening line is already noted in the section on GSEA, however we agree with the reviewer and have simply deleted it and added a partial line of further down to highlight how these observations tie in with previous studies.

Lines 471-474: These lines mention how interesting it was to identify impacted processes through GSEA. This statement supports my point above that some of the info in Supplemental file 3 should be included in the main text. You have nice, informative data; please make it more pronounced!

Agreed, and it is now included.

Supplemental file 1: missing from the submission, but not critical for the review. Please make sure it is accessible to readers.

It is actually fairly critical as some comments above would have not been needed if the reviewer had been able to download the file.

Table 6: This table could be greatly improved by adding some data to it. Specifically, the reader would benefit from seeing the fold change in expression for each gene in the different treatments and years.

The data is too important to be left buried in the supplemental files. I think this just means adding two additional columns, one for 2007 fold change and one for 2008 fold change.

We agree and the additional data was added to the table.

Minor Points

Lines 125-129: What is the source of the 30-year normal data, and how was GDD calculated. Please be specific.

We used 30 year normal data from the degree day tool at https://climate.sdstate.edu, which calculates precipitation and temperature data. At the time of calculation, 30 year normals were presented along with other data. GDD were calculated utilizing the 86/50 system. In short, daily high temp (up to 86F) was added to the low temp (anything lower than 50F is calculated as 50), the sum is divided by 2, and 50 is then subtracted to get total GDD. Line 126 now reads "…accumulated growing degree days (GDD, using the 86/50 system)…" Table 1 header has been modified to indicate source of data as well.

Line 140: canola was planted at a rate of 7 kg ha-1. Please translate this value to the number of weeds per row, as was done for velvetleaf. Or, find another way to describe the weed pressure with common terms/units so that the reader can better understand the weed pressure in the 2 years.

Medium size canola seed average 250,000 seeds/kg. At the generally recognized emergence rate of 55%, this calculates out to 96 plants/m^2. Lines 139-143 now read: " In 2008, another broadleaf, canola (*Brassica napus*), was drilled 10 cm from the maize row at 175 seeds /m^2 at maize planting to provide a more uniform 'weed' density (anticipating a 55% emergence rate, or 96/m^2 average weed density)."

Lines 145-147: "Weed-stressed plots..." Please specify WR8 plots. I think this is what is meant. Also, on line 147, was weed pulling also after tissue sampling? Please specify.

We did mean the V4 stage here where weeds were removed with an additional roundup treatment at about ½ field strength. Also, we confirmed that weeds were not removed prior to harvest at V8, but the WR4 and WR8 plots were kept weed free thereafter by hand techniques. The sentence now reads "weeds were mechanically controlled using a hoe and hand pulling after data and sample collection at V8 until harvest."

Line 158: Please indicate which leaf was used for SPAD readings.

We used the most recently collared leaf for SPAD readings, and have modified the text in line 158 to clarify.

Line 195: Please contact the Gene Expression Omnibus to make sure the expression data is released to the public. It is currently private until June 15, 2019.

Odd… I almost always make data available for immediate release. In any case, I went into GEO and released this data.

Line 226 and Line 261: Please indicate which version of the maize reference genome was used for mapping reads.

Corrected to include the *Zea mays* Ensemble 19 reference genome.

Lines 354-355: The statement that glyphosate isn't a factor because of when it was applied does not make sense when considering that a large part of this manuscript is about weed pressure having an effect long after the weeds were removed. I suggest removing that speculation and instead base your argument on the studies that you cite. (don't add anything, just remove that statement).

We are going to leave this statement in because it was added to address a point of contention noted by an earlier review of this manuscript.

Line 390: "WF" is used for the first time. I assume this means weed free, which is equivalent to control used in some tables. So, WF=weed free=control. Would be best to pick one term and be consistent. Supplemental file 3: all tabs in the file are labeled 2 instead of 3. This was a little confusing, please correct it.

We removed mention of WF and now only use the term “weed free control” throughout the manuscript

Figure 1 legend: line 147 says that weeds were manually removed after V8, but the legend says that herbicides were used. Which is it?

I see where there was confusion. This now reads “The weed free control plots were treated with herbicides for weed control prior to the critical weed free period (CWFP) at V2 and again several weeks

prior to V8, WR4 plots were treated at the V4 stage for weeds removal at that stage; no herbicide was applied to weedy (WR8) treatments until after data and plant tissue collection at V8."

Table 4: please indicate in the legend (or M&M) whether reads mapped to the reference genome or the reference transcriptome. Also, indicate the version.

For Tophat, and as noted in the MM mapping of reads was to the *Zea mays* Ensemble 19 reference genome. For the de novo assembly, reads were mapped back to the Trinity-generated reference transcriptome (now specifically stated in the MM).
